# Titanium Allergy: A Case of Foreign Body Reaction Following Laparoscopic Cholecystectomy

**DOI:** 10.7759/cureus.104781

**Published:** 2026-03-06

**Authors:** Almir Music, Abhishek A Thapa, Mariia Shipovskaia, Ashiq Pramchand, Bradley Moushon, Frederick Tiesenga

**Affiliations:** 1 Department of General Surgery, Washington University of Health and Science, San Pedro, BLZ; 2 Department of General Surgery, Suvekchya International Hospital, Kathmandu, NPL; 3 Department of General Surgery, Northwestern University Feinberg School of Medicine, Chicago, USA; 4 Department of General Surgery, University of KwaZulu-Natal, Nelson R. Mandela School of Medicine, Durban, ZAF; 5 Department of General Surgery, St. George’s University School of Medicine, St. George’s, GRD; 6 General Surgery, West Suburban Medical Center, Chicago, USA

**Keywords:** biocompatibility, delayed hypersensitivity, foreign bodies, laparoscopic cholecystectomy (lc), metal hypersensitivity, postcholecystectomy syndrome, surgical instruments, titanium clips

## Abstract

Titanium is widely regarded as a biocompatible and inert material commonly used in surgical implants. Despite this reputation, hypersensitivity reactions to titanium, though rare, can lead to persistent and unexplained postoperative symptoms. We report the case of a 76-year-old woman who developed progressive right upper quadrant abdominal pain, burning sensations, neuropathic symptoms, and fatigue three months after laparoscopic cholecystectomy in which titanium clips were used.

Her history included documented systemic intolerance to a titanium dental implant 10 years earlier, which had required removal. Extensive laboratory and imaging evaluation was unremarkable. Titanium hypersensitivity was clinically suspected based on the temporal association between clip placement and symptom onset, along with systematic exclusion of alternative causes. MELISA® (memory lymphocyte immunostimulation assay) testing was discussed, but declined due to cost and extended turnaround time. Following diagnostic laparoscopy and removal of the four titanium clips approximately three months after symptom onset, the patient experienced complete symptom resolution within one week. She remains asymptomatic at six-month follow-up.

This case highlights the importance of considering metal hypersensitivity in patients with unexplained postoperative symptoms and prior exposure to implants. Increased awareness and selective preoperative screening, particularly evaluation for prior metal intolerance, may help prevent unnecessary morbidity and repeated interventions.

## Introduction

Titanium hypersensitivity is a rare but increasingly recognized cause of persistent postoperative symptoms, particularly in patients with prior metal exposure. Although titanium is widely regarded as biocompatible and inert, delayed type IV hypersensitivity reactions have been documented in susceptible individuals [1-4]. Reported prevalence ranges from approximately 0.2% to 3% in the general population, with higher rates observed among those previously exposed to titanium-containing implants [1,5]. Despite its rarity, under-recognition may contribute to prolonged diagnostic evaluation and unnecessary patient morbidity.

Post-cholecystectomy syndrome (PCS) refers to the persistence or recurrence of right upper quadrant (RUQ) pain or gastrointestinal symptoms following gallbladder removal, often resembling preoperative complaints [6]. Common manifestations include fatty food intolerance, nausea, vomiting, indigestion, diarrhea, jaundice, and abdominal pain [7]. The differential diagnosis of PCS is broad and encompasses biliary, extra-biliary, functional, and idiopathic causes [1]. In rare cases, hypersensitivity to surgical materials such as titanium clips may clinically mimic PCS [5].

Laparoscopic cholecystectomy is one of the most commonly performed abdominal procedures and routinely utilizes titanium clips for cystic duct and artery ligation due to their strength, corrosion resistance, MRI compatibility, and ease of application [8-10]. Although generally considered safe, implant-related metal hypersensitivity reactions have been reported and often present with vague, nonspecific symptoms that complicate diagnosis [2-4].

This report describes a rare case of titanium clip hypersensitivity following laparoscopic cholecystectomy in a patient with a documented history of titanium dental implant intolerance. By contributing to the limited body of published cases [11,12], this case aims to increase awareness of this under-recognized complication and highlight the importance of clinical suspicion in patients with unexplained postoperative symptoms.

## Case presentation

A 76-year-old woman presented with a three-month history of progressive fatigue, decreased appetite, and RUQ pain described as burning and radiating to the left arm and thumb. At presentation, she rated her RUQ pain as 7/10 in severity. She also reported nausea, worsening constipation, and neuropathic discomfort involving the epigastric region and torso. These symptoms began after a laparoscopic cholecystectomy in February 2025, during which four titanium clips were applied for cystic duct and artery ligation.

Her prior medical history was notable for systemic intolerance to a titanium dental implant approximately 10 years earlier, characterized by fatigue, gingival swelling, headaches, and functional limitations, which resolved completely following implant removal. She had no chronic medical conditions, took no regular medications, and denied smoking, alcohol, or substance use. Titanium was the only suspected allergen.

Physical examination demonstrated mild RUQ tenderness without peritoneal signs or other abnormalities. Hematologic, hepatic, and inflammatory laboratory evaluations were within normal limits (Table 1). Abdominal ultrasound and computed tomography imaging demonstrated no acute pathology, retained stones, biliary dilation, or structural complications.

**Table 1:** Comprehensive preoperative laboratory results Baseline hematologic indices, hepatic function parameters, and inflammatory markers were within normal reference ranges. There was no laboratory evidence of leukocytosis, cholestasis, hepatocellular injury, or systemic inflammatory response. These findings supported exclusion of alternative biliary, infectious, or inflammatory etiologies contributing to the patient’s persistent post-cholecystectomy symptoms.

Laboratory Test	Analyte (Abbreviation)	Result	Reference Range
Complete blood count (CBC)	Leukocytes (WBC)	5.6 ×10⁹/L	4.0–11.0 ×10⁹/L
	Erythrocytes (RBC)	4.78 ×10¹²/L	3.80–5.00 ×10¹²/L
	Hemoglobin (Hgb)	140 g/L	120–150 g/L
	Hematocrit (Hct)	0.41 L/L	0.34–0.45 L/L
	Mean corpuscular volume (MCV)	86.6 fL	80.0–97.0 fL
	Mean corpuscular hemoglobin (MCH)	29.3 pg	27.0–33.0 pg
	Mean corpuscular hemoglobin concentration (MCHC)	338 g/L	320–360 g/L
	Red cell distribution width (RDW)	13.5%	12.0–15.0%
	Platelets (Plt)	215 ×10⁹/L	140–400 ×10⁹/L
	Mean platelet volume (MPV)	9.3 fL	8.0–13.0 fL
CBC differential	Neutrophils	2.78 ×10⁹/L	1.50–8.00 ×10⁹/L
	Lymphocytes	2.28 ×10⁹/L	0.50–3.50 ×10⁹/L
	Monocytes	0.45 ×10⁹/L	0.20–0.90 ×10⁹/L
	Eosinophils	0.10 ×10⁹/L	0.00–0.50 ×10⁹/L
	Basophils	0.02 ×10⁹/L	0.00–0.10 ×10⁹/L
	Immature granulocytes	0.01 ×10⁹/L	0.00–0.07 ×10⁹/L
Hepatic function panel	Total bilirubin	14 µmol/L	2–18 µmol/L
	Alkaline phosphatase (ALP)	66 U/L	34–104 U/L
	Alanine aminotransferase (ALT/SGPT)	16 U/L	<38 U/L
Inflammatory marker	High-sensitivity C-reactive protein (hs-CRP)	0.99 mg/L	<3.0 mg/L

Given the patient’s prior implant reaction and persistent unexplained postoperative symptoms despite normal laboratory and imaging findings, titanium hypersensitivity was clinically suspected. MELISA® (memory lymphocyte immunostimulation assay) testing was discussed in light of the prior titanium dental implant intolerance, but was deferred due to limited availability, cost considerations, and prolonged processing time. Diagnostic laparoscopy with targeted foreign body removal was performed three months after the index cholecystectomy.

**Figure 1:**
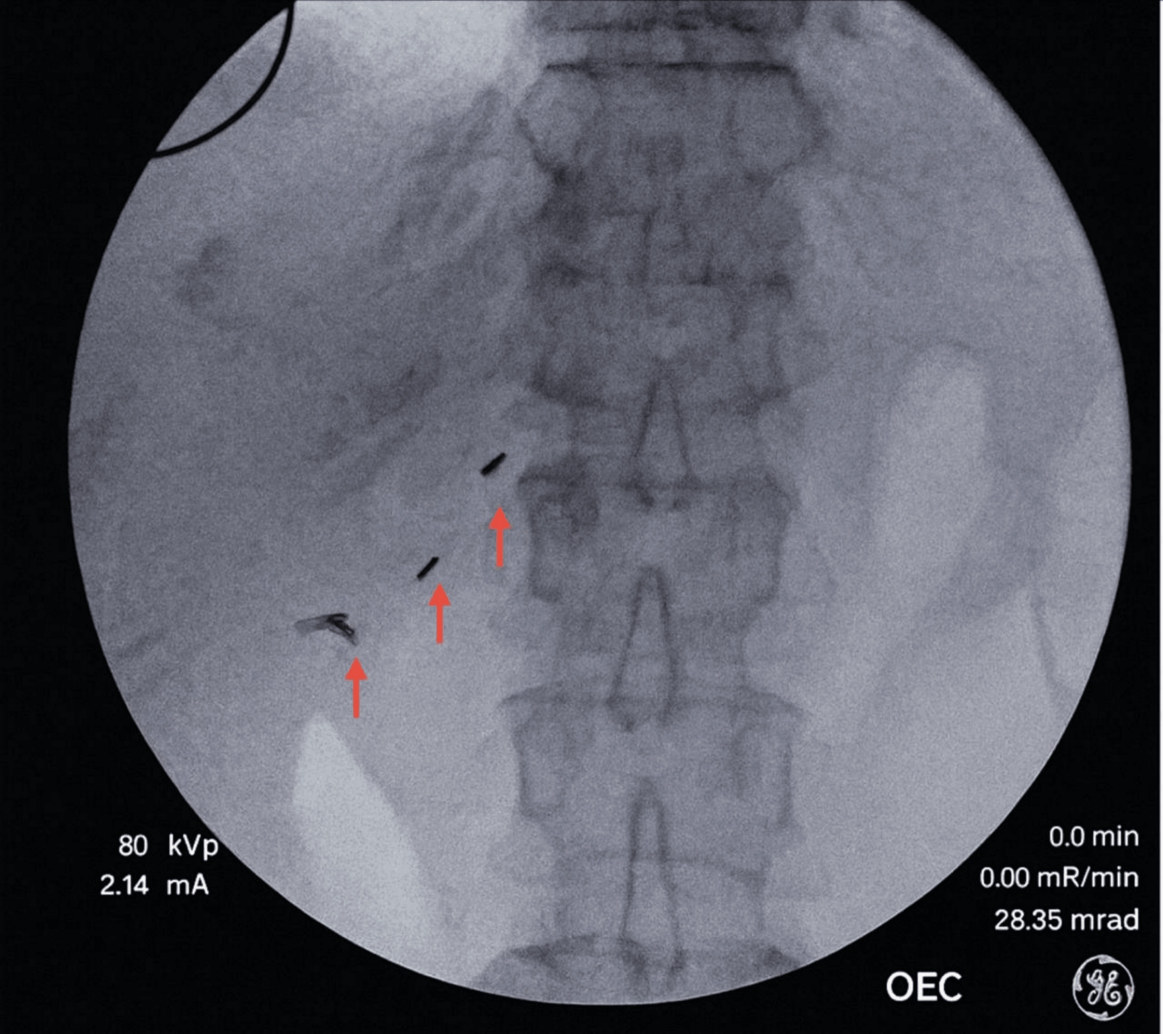
Intraoperative fluoroscopic localization of retained titanium surgical clips in situ prior to removal The intraoperative C-arm fluoroscopic image obtained during diagnostic laparoscopy demonstrates multiple radiopaque metallic densities (red arrows) consistent with retained titanium surgical clips along the right upper quadrant in the region of the prior cystic duct and artery ligation. The clips are visualized embedded within fibrotic tissue at the previous operative site. Fluoroscopic guidance facilitated precise localization and targeted dissection, allowing removal while minimizing additional tissue trauma. This imaging confirms the presence of metallic foreign bodies prior to explantation.

Intraoperative fluoroscopy confirmed the presence of retained titanium clips embedded within fibrotic tissue (Figure 1).

Surgical procedure

**Figure 2:**
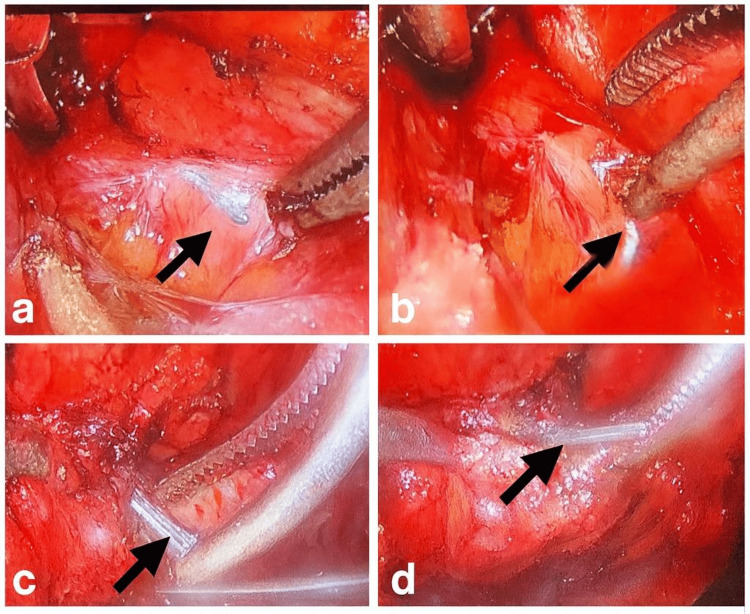
Sequential intraoperative laparoscopic images demonstrating dissection and mobilization of a titanium clip embedded within dense fibrotic tissue (a) The initial laparoscopic view of the prior cystic duct and artery ligation site demonstrates dense fibrotic scarring and adhesions along the dorsal peritoneal surface. The metallic titanium clip (arrow) is partially visualized beneath overlying fibrotic tissue, reflecting incorporation within the postoperative scar. (b) Sharp and blunt dissection was performed carefully to separate fibrotic adhesions and expose the embedded clip. Progressive exposure highlights the extent of tissue integration and the need for controlled, stepwise dissection to avoid injury to adjacent structures. (c) Clear delineation of the titanium clip (arrow) within dense adhesions following further mobilization. This image illustrates the technical complexity of retrieval in a previously operated field characterized by scarring and altered anatomy. (d) Final mobilization of the clip (arrow) prior to extraction. The clip is fully exposed and prepared for removal while preserving the integrity of the cystic duct stump, vascular structures, and surrounding biliary anatomy.

During laparoscopy, four titanium clips deeply embedded within dense fibrotic tissue along the dorsal peritoneal surface were identified. Sequential dissection revealed the clips adherent within scar tissue, requiring meticulous sharp and blunt dissection to avoid injury to adjacent biliary and vascular structures (Figure 2). The images demonstrate the technical complexity of clip retrieval in a previously operated field.

**Figure 3:**
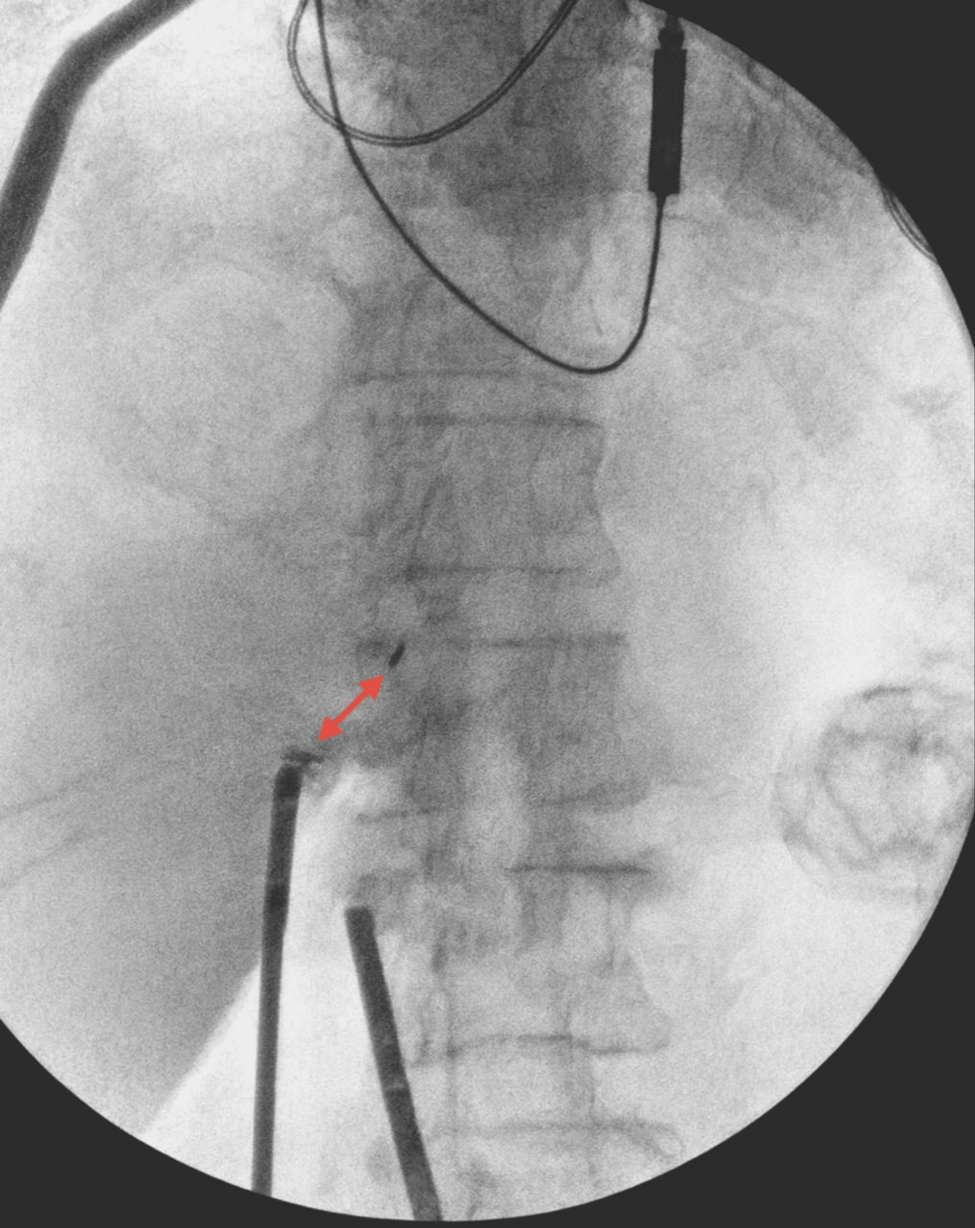
Intraoperative C-arm fluoroscopic guidance during titanium clip retrieval An intraoperative fluoroscopic image obtained during active dissection demonstrates radiopaque metallic clips (double-headed arrow). Surgical instruments are visible within the operative field, illustrating real-time C-arm guidance used to localize the clip during retrieval. Fluoroscopy facilitated precise spatial orientation relative to surrounding anatomy and supported controlled extraction while minimizing additional dissection in a previously scarred surgical bed. This image demonstrates the utility of intraoperative imaging for targeted foreign body removal when clips are embedded within fibrotic tissue and not easily identifiable by direct visualization alone.

Under fluoroscopic C-arm guidance, all four clips were precisely localized and removed with minimal additional dissection (Figure 3). Real-time imaging facilitated targeted extraction while minimizing unnecessary tissue trauma. Inspection confirmed that the cystic duct and artery stumps remained intact and secure, indicating that only the redundant foreign material was removed without compromising prior ligation.

The postoperative course was uneventful. The patient required no analgesics, antibiotics, corticosteroids, antihistamines, or other adjunctive medical therapy for symptom control following clip removal. Within one week postoperatively, she reported complete resolution of RUQ pain, fatigue, and neuropathic symptoms, with restoration of appetite and baseline functional status. Her pain improved from 7/10 preoperatively to 0/10 within one week of clip removal. There were no postoperative complications and no recurrence of symptoms at standard surgical follow-up.

**Figure 4:**
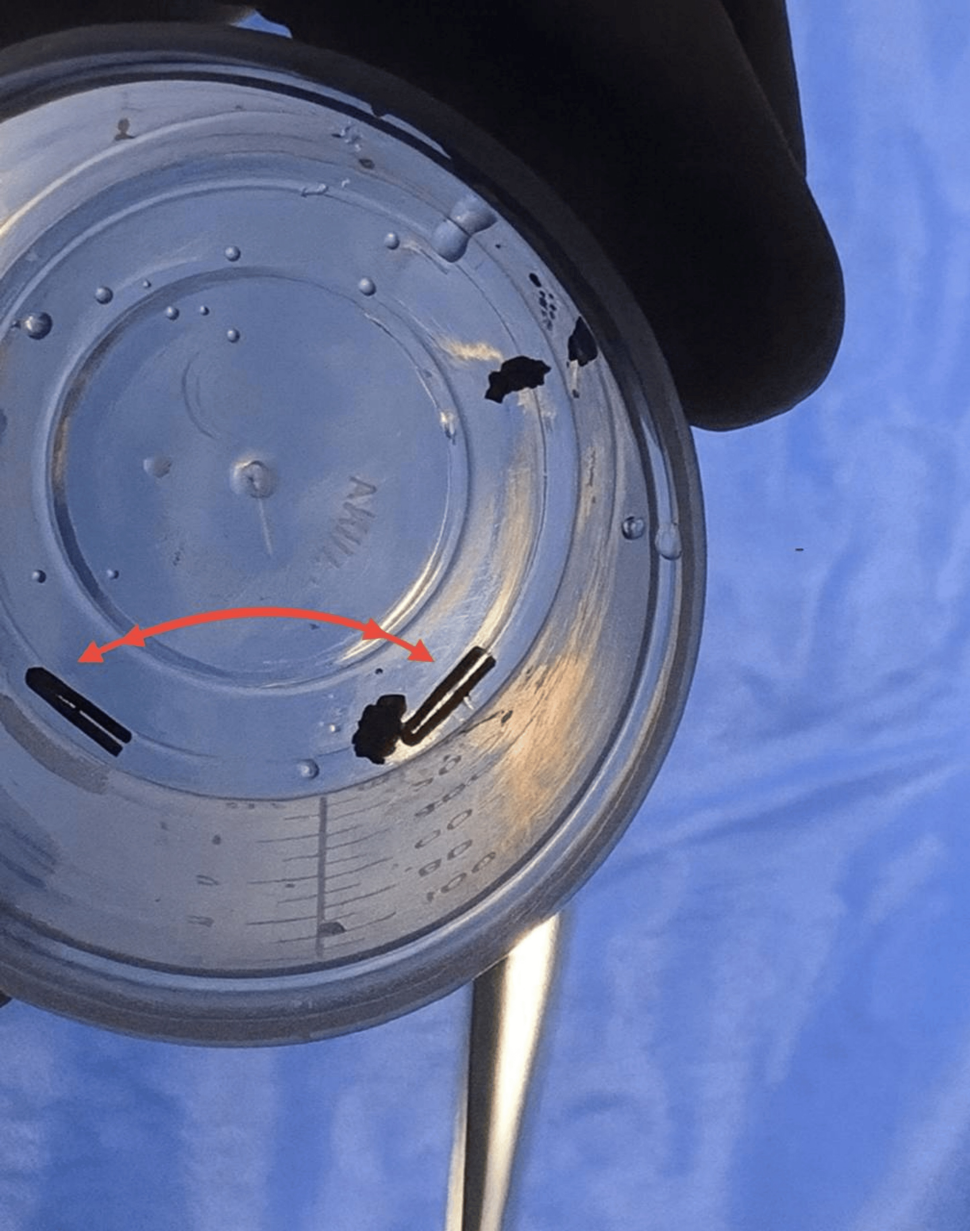
Retrieved titanium surgical clips following explantation A specimen photograph demonstrates two of the four titanium surgical clips removed during diagnostic laparoscopy. The retrieved clips are shown after complete extraction from the prior cystic duct and artery ligation site. This image provides direct confirmation of foreign body removal and correlates temporally with the patient’s complete postoperative symptom resolution.

Two of the retrieved titanium clips are shown in Figure 4, providing direct visual confirmation of foreign body removal.

## Discussion

Hypersensitivity reactions represent exaggerated or inappropriate immune responses to foreign antigens and are classically categorized into four immunologic types (Table 2) [13].

**Table 2:** Categories of hypersensitivity reactions This table provides an overview of the four major categories of hypersensitivity reactions, outlining their underlying immune mechanisms, characteristic clinical manifestations, and typical onset patterns. This classification system highlights the spectrum of immune responses, ranging from rapid IgE-mediated reactions to delayed T-cell–driven inflammation. Understanding these categories is clinically important, as they serve as a framework for recognizing diverse presentations in surgical and postoperative settings. Adapted from Warrington et al. (2011) [13].

Type	Immune Basis	Examples of Clinical Presentation	Time Frame of Onset
I	IgE-driven immediate reaction	Rapid onset of anaphylaxis, hives, airway constriction, or soft tissue swelling	Within minutes to a few hours
II	Antibody-mediated cytotoxicity (IgG/IgM)	Hemolysis, platelet destruction, transfusion-related complications	Usually hours to several days
III	Immune complex–driven inflammation	Vasculitic rashes, kidney inflammation, serum sickness–like illness	Hours to days after exposure
IV	Delayed T-cell–mediated response	Contact dermatitis, chronic granulomatous inflammation	Typically 2–3 days after exposure

While titanium is widely regarded as biocompatible and corrosion resistant, increasing evidence suggests that it can, in rare instances, provoke delayed type IV hypersensitivity reactions [4-5,8-9]. These reactions are mediated by T-cell activation and may clinically manifest with localized inflammation, systemic fatigue, neuropathic symptoms, or nonspecific constitutional complaints [13,14].

In the postoperative setting, such presentations are frequently misattributed to more common biliary etiologies, particularly post-cholecystectomy syndrome, and clinicians should remain mindful of atypical postoperative symptom patterns suggestive of material intolerance [6,15]. PCS typically presents with upper abdominal pain, dyspepsia, indigestion, or diarrhea following gallbladder removal [1]. However, systemic manifestations such as diffuse burning pain, neuropathic symptoms, or unexplained fatigue are not characteristic of classic PCS and should prompt consideration of alternative diagnoses, including hypersensitivity to surgical materials.

Titanium clips are favored in laparoscopic cholecystectomy due to their strength, corrosion resistance, MRI compatibility, and overall biocompatibility [10]. They are widely used across multiple surgical specialties, including general surgery, orthopedics, neurosurgery, cardiovascular surgery, and dentistry. Although nickel allergy is more commonly reported in the general population (up to 6%-8%) [4], titanium hypersensitivity appears comparatively rare and likely under-recognized. The available literature suggests that true immunologic cross-reactivity between titanium and other metals, such as nickel, is limited, with reported reactions more often representing independent sensitizations rather than classic cross-reactive mechanisms [1,14]. This underreporting may contribute to prolonged diagnostic delays, during which patients undergo extensive hematologic, radiologic, and sometimes psychiatric evaluation, often at significant financial and emotional costs [8,9].

Differentiating PCS from metal hypersensitivity

Although both PCS and metal hypersensitivity may present with abdominal discomfort, key distinctions exist. PCS is typically related to biliary, structural, or functional causes and does not involve systemic immunologic activation. In contrast, metal hypersensitivity reactions may include constitutional symptoms such as fatigue, neuropathic pain, diffuse burning sensations, or delayed inflammatory responses in the absence of structural abnormalities. Recognition of these distinguishing features is essential to prevent unnecessary investigations and to expedite definitive management.

In patients with a suggestive history of metal intolerance, selective preoperative evaluation may be considered, including assays such as MELISA® where available [16,14]. However, confirmatory testing remains imperfect, and diagnosis often relies on clinical suspicion, exclusion of alternative causes, and documented symptom resolution following removal of the suspected implant [14]. Routine preoperative screening is not recommended in the absence of a documented history of metal intolerance, given the rarity of clinically significant titanium hypersensitivity.

Our patient’s case underscores this diagnostic complexity. Her prior systemic intolerance to a titanium dental implant, recognized retrospectively as clinically relevant, provided biologic plausibility for recurrence of similar symptoms following titanium clip placement. The temporal association between clip implantation and symptom onset, followed by complete resolution after explantation, supports a likely causal relationship.

Similar clinical improvement after removal of metallic surgical clips has been reported in the literature [1,8-9], reinforcing the need for heightened clinical awareness in patients presenting with persistent unexplained postoperative symptoms.

Overall, although uncommon, titanium hypersensitivity should remain within the differential diagnosis in patients with atypical or systemic symptoms after implant-based procedures. Increased recognition of this entity may reduce diagnostic delays, promote early diagnosis, facilitate timely, targeted intervention, and potentially reduce unnecessary investigations, morbidity, and additional surgeries [17].

## Conclusions

Although uncommon, titanium hypersensitivity is clinically relevant and should be considered in patients presenting with persistent or atypical postoperative symptoms, particularly in those with a documented history of intolerance to metal implants. This case illustrates that titanium clips, while generally regarded as safe and biocompatible, may contribute to systemic or neuropathic symptoms that resolve following removal. Careful perioperative evaluation, including attention to prior adverse reactions to metallic implants, is essential to reducing diagnostic delay.

When clinical suspicion arises, selective diagnostic testing such as patch testing, MELISA®, or lymphocyte transformation assays may be considered in appropriate patients, recognizing the current limitations in standardization and diagnostic sensitivity. In higher risk individuals, alternative fixation strategies, including absorbable sutures or polymer-based clips, may offer effective surgical management without compromising procedural outcomes. Importantly, the absence of validated diagnostic criteria and standardized screening protocols remains a significant limitation in accurately identifying and quantifying titanium hypersensitivity. Future multicenter studies, collaborative registry reporting, and the development of standardized diagnostic frameworks are necessary to better define incidence rates, clarify pathophysiologic mechanisms, and guide evidence-based perioperative decision-making. Increased awareness among surgeons and clinicians may facilitate earlier recognition, reduce unnecessary investigations and hospitalizations, and ultimately improve postoperative quality of life for affected patients.
